# The effect of Parkinson’s disease on total knee arthroplasty: a systematic review and meta-analysis

**DOI:** 10.1186/s43019-023-00179-1

**Published:** 2023-02-14

**Authors:** Jung-Ro Yoon, Tae-Hyuck Yoon, Seung Hoon Lee

**Affiliations:** Department of Orthopedic Surgery, Veterans Health Service Medical Center, 53 Jinhwang-do-ro 61-gil, Gangdong-gu, 05368 Seoul, Korea

**Keywords:** Total knee arthroplasty, Parkinson’s disease, Clinical outcome, Complication

## Abstract

**Purpose:**

The purpose of this systematic review was to determine the effect of Parkinson’s disease (PD) on clinical outcomes and complications after total knee arthroplasty (TKA). Our systematic review was conducted to answer the following questions: (1) does TKA negatively affect clinical outcomes in patients with PD? and (2) does TKA cause more complications in patients with PD?

**Methods:**

A rigorous and systematic approach was used, and each selected study was evaluated for methodological quality. Data on study design, total number of cases enrolled, follow-up duration, PD severity, clinical outcome, and complications after TKA were analyzed.

**Results:**

Fourteen studies were included. Nine studies reported clinical scores. TKA significantly increased knee and functional scores in the PD group. However, compared with knee and functional scores in the non-PD group, the increase in scores in the PD group was not statistically significant, but tended to be less than that in the non-PD group. Eleven studies reported complications. In six studies, there was no difference in the complication rate between the PD and non-PD group or did not include a control group. In five studies, the PD group had higher medical complication rates and similar or higher surgical complication rates than the non-PD group.

**Conclusions:**

Patients with PD who underwent TKA showed satisfactory functional improvement and pain reduction. However, these outcomes were not as good as those in the non-PD group. The PD group had a higher probability of occurrence of medical complications than the non-PD group. Further, the PD group had a similar or higher surgical complication rate than the non-PD group.

## Introduction

Total knee arthroplasty (TKA) is a common surgery for end-stage knee osteoarthritis (OA) treatment and can improve patients’ pain and function. In recent years, due to an increase in the life expectancy, many cases of TKA have been reported in elderly patients, which in turn has increased the number of patients with underlying diseases requiring TKA [1, 2]. Parkinson’s disease (PD) is a common underlying disease in elderly individuals. PD is the second most common neurodegenerative disease, with approximately 2% men and 1.3% women having a lifetime risk of PD [3]. The incidence of PD is low at a young age, but increases with age and is the highest at the age of 80 years [4].

PD is a neurodegenerative disease that exhibits characteristic clinical symptoms such as rigidity, bradykinesia, and reduced amplitude and automaticity of movement [5]. Gait impairment is a clinical manifestation of PD; its severity worsen with disease progresses [6]. Gait impairment is a problem that degrades quality of life. PD may also increase the risk of osteoporosis and falls [7]. Therefore, patients with PD and knee OA undergoing TKA may have poor outcomes.

Due to the characteristics of PD, patients undergoing TKA may have more postoperative complications and decreased functional outcomes. In addition, patients with PD have higher rates of mortality and medical comorbidity than normal patients [8]. Flexion contracture progresses as PD progresses, which adversely affects the outcome of TKA [9]. PD can also affect perioperative complications and patient satisfaction after TKA [10]. Moreover, TKA cannot prevent the progression of PD, because TKA improves only the mechanical problems of the knee. However, this does not mean that the patients are not conductive to pain reduction and functional improvement after surgery. Many studies have reported that TKA is helpful in improving the function and symptoms after the procedure.

In general, when selecting TKA as a treatment for knee OA, pain reduction, functional improvement, and complications after TKA are considered. PD can affect TKA outcomes, and several studies have reported the effect of PD on TKA. Many studies have compared and reported TKA outcomes in patients with PD, but the results are inconsistent, possibly due to the varying severity of PD and extent of complications reported in the study [11, 12]. Therefore, the purpose of this systematic review and meta-analysis was to determine the effect of PD on the clinical outcomes and complications after TKA. Our systematic review and meta-analysis was conducted to answer the following questions by analyzing studies that assessed TKA in patients with PD: (1) does TKA negatively affect clinical outcomes in patients with PD? and (2) does TKA cause more complications in patients with PD?

## Materials and methods

### Search strategy

To verify the research question, a rigorous and systematic approach conforming to the Preferred Reporting Items for Systematic Review and Meta-Analysis (PRISMA) guidelines was used [13]. In phase 1 of the PRISMA search process, selected databases were searched for eligible articles, including the MEDLINE, EMBASE, and Cochrane databases (30 December 2022). A Boolean strategy was used, and the field search terms included the following: (“primary total knee arthroplasty” or “total knee arthroplasty” or “primary total knee replacement” or “total knee replacement”) and (“Parkinson” or “Parkinson’s disease” or “Parkinson disease”). The citations in the included studies were screened, and unpolished articles were manually checked. The bibliographies of the relevant articles were subsequently cross-checked for articles not identified in the search. In phase 2, abstracts and titles were screened for relevance. In phase 3, the full text of the selected studies was reviewed according to the inclusion criteria, and methodological appropriateness was determined using a predetermined question. In phase 4, the studies were subjected to a systematic review process, if appropriate.

### Eligibility criteria

Studies meeting the following criteria were included: (1) studies on TKA and PD, (2) articles written in English, (3) articles with full text available, and (4) articles including clinical outcomes or complications. The exclusion criteria were as follows: (1) studies not related to TKA, (2) studies published before 2000, (3) meta-analysis or systematic review articles, and (4) studies written in language other than English.

### Data extraction

Each of the selected studies was evaluated for methodological quality by two independent authors. Data were extracted using the following standardized protocol: first author, publication year, publication journal, study type, number of cases, follow-up period, preoperative and postoperative clinical and functional scores, and complications. The extracted data were then cross-checked for accuracy, and any disagreements were resolved by a third author.

### Quality assessment

The methodological quality of cohort studies or nonrandomized case–control studies was assessed using the Newcastle–Ottawa assessment scale [14]. This consists of three main domains (selection, comparability, and outcome), with four categories in the selection domain, one category in the comparability domain, and three categories in the outcome domain. A study was awarded a maximum of one or two stars for each item within the selection and outcome domains. A maximum of two stars was given for comparability. More stars indicated lower risk of bias.

### Statistical analysis

Statistical analysis was performed for knee scores and functional scores using Stata version 14.2 static software. All the results are presented as forest plots. The 95% confidence interval (CI) was calculated for each effect size. The *I*^2^ statistic, which shows the percentage of total variation attributable to the heterogeneity among studies, was calculated, and values of < 25%, 50%, and > 75% were interpreted as small, moderate, and high levels of heterogeneity, respectively. A random effects model rather than a fixed effects model was used to calculate the effect size, as it was assumed that studies within each subgroup did not share a common effect size.

## Results

### Search

**Fig. 1:**
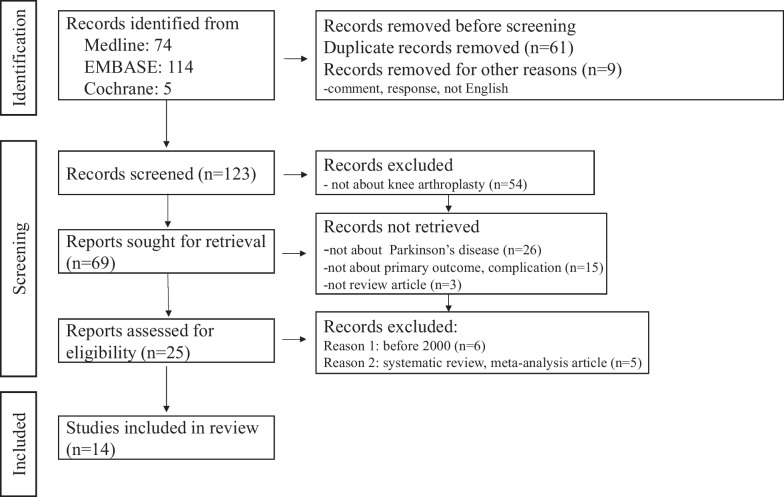
PRISMA flowchart

An initial electronic search yielded 193 articles. After excluding duplicate studies and applying the inclusion and exclusion criteria, 14 articles were included in the final analysis (Table 1). Some articles used registry data, some included retrospective cohorts, some enrolled prospective cohorts, and some were case–control studies. The PRISMA flowchart is shown in Fig. 1.

**Table 1:** Characteristics of the included studies

Journal	Year	Author	Study design	Level of evidence	Disease severity	Follow-up duration	Number (PD)	Number (control)	Age in years
Acta Orthop. Belg	2013	Craig	Retrospective case-controlled study	III	No information	5 years	32	33	Mean 73
JBJS	2014	Jämsen	Registry-based case-controlled study	III	No information	PD: median 5.4 years Control: median 5.5 years	560	1680	PD: median 72 Control: median 73
J Arthroplasty	2017	Rodon	Retrospective case-controlled study	III	Charlson Comorbidity index PD: 3.72 ± 0.14 Control: 3.84 ± 0.12	5.3years	71	132	PD: 68.5 ± 1.13 Control: 69.7 ± 0.77
J Arthroplasty	2018	Wong	Retrospective cohort study	III	No information	1 year	35	41	PD: 72.6 ± 7.3 Control: 71.8 ± 7.6
J Orthop Surg Res	2019	Xiao	Retrospective case series	IV	Hoehn and Yahr stage I: 2, II: 7, III: 5, IV: 4	Median 38 months	18		67.89 ± 6.62
KSSTA	2019	Newman	Registry-based case-controlled study	III	Charlson Comorbidity index PD: 0: 59% (18,928), 1: 26% (8288), ≥ 2: 15% (4705) Control: 0.59% (56,707), 1: 26% (24,827), ≥ 2: 15% (14,062)		31,921	95,596	PD: 72 ± 8.1 Control: 72 ± 8.1
Knee	2019	Kleiner	Registry-based case-controlled study	III	Charlson Comorbidity index PD: 0.56 ± 0.01 Control: 0.58 ± 0.00		7361	73,610	PD: 72.0 ± 0.1 Control: 72.1 ± 0.03
AOTS	2020	Veronica	Retrospective cohort study	IV	Modified Hoehn and Yahr stage 1.5	3.5 years	26		71 (range 61–83)
JKS	2020	Marchand	Registry-based case-controlled study	III	Elixhauser Comorbidity index PD: 9 ± 4 Control: 9 ± 4	2 years	18,082	54,244	< 64: PD (2255), Control (6765) 65–69: PD (4740), Control (14,220) 70–74: PD (4483), Control (13,448) 75–79: PD (3867), Control (11,601) 80–84: PD (2008), Control (6024) < 85: PD (565), Control (4695) Unknown: PD (164), Control (491)
J Arthroplasty	2020	Goh	Retrospective case-controlled study	III	Hoehn and Yahr stage I: 34, II: 12, III: 9, IV: 2	2 years	57	57	PD: 69.3 ± 7.7 Control: 70.1 ± 8.6
J Orthop Sci	2020	Ergin	Retrospective case-controlled study	III	Columbia stage in PD I: 2, II: 7, III: 4	PD: 64.5 ± 44.7 months Control: 51.8 ± 13.6 months	13	13	PD: 75.6 ± 8.13 Control: 71.4 ± 9.07
Int Orthop	2021	Baek	Retrospective case-controlled study	III	Koval’s grade PD: I (25), II (4) Control: I (51), II (7)	PD: mean 133.0 months Control: mean 133.1 months	29	58	PD: 71.0 ± 5.8 Control: 71.2 ± 5.6
BMC Musculoskeletal Disorders	2022	Y. Zong	Retrospective case-controlled study	III	Hoehn and Yahr stage 2.3 ± 0.9	PD: 13.8 ± 7.2 months Control: 14.5 ± 8.3 months	12	48	PD: 65.4 ± 11.4 Control: 65.2 ± 10.9
J of AAOS	2022	Cheppalli	Registry-based case-controlled study	III	No information	4 years	3082	555,289	PD: 71.44 ± 7.88 Control: 66.59 ± 9.50

### Quality

The quality assessment details are presented in Table 2. Twelve case–control studies and two cohort studies were assessed using the Newcastle–Ottawa assessment scale. Among the 12 case–control studies, six studies were awarded four stars, five studies were awarded three stars, and one study was awarded two stars of a possible four stars in the selection domain. In the comparability domain, all studies were awarded one star. In the outcome domain, five studies were awarded four stars, six studies were awarded three stars, and one study was awarded one star of a possible four stars. Both cohort studies showed a low risk of bias in all three domains. One was awarded five stars, and one was awarded three stars of a possible six stars in the selection domain. In the comparability domain, all studies were awarded one star. In the outcome domain, one study was awarded four stars and one study was awarded two stars of a possible five stars.

**Table 2:** Quality assessment of the included studies using Newcastle–Ottawa assessment

Author	Journal	Year	Criteria							
			Selection				Comparability	Outcome		
*Case–control study*			(1) (*)	(2) (*)	(3) (*)	(4) (*)	(1) (**)	(1) (**)	(2) (*)	(3) (*)
Craig	Acta Orthop. Belg	2013	*	*		*	*	**	*	*
Jämsen	JBJS	2014	*	*	*	*	*	*	*	*
Rodon	J Arthroplasty	2017	*	*		*	*	**	*	*
Newman	KSSTA	2019	*	*	*	*	*	*	*	*
Kleiner	Knee	2019	*	*	*	*	*	*	*	*
Xiao	J Orthop Surg Res	2019	*	*			*	*		
Marchand	JKS	2020	*	*	*	*	*	*	*	*
Goh	J Arthroplasty	2020	*	*		*	*	**	*	*
Ergin	J Orthop Sci	2020	*	*		*	*	**	*	*
Baek	Int Orthop	2021	*	*	*	*	*	**	*	*
Y. Zong	BMC Musculoskeletal Dis	2022	*	*		*	*	*	*	*
Cehppalli	J of AAOS	2022	*	*	*	*	*	*	*	*
*Chort study*			(1) (**)	(2) (*)	(3) (**)	(4) (*)	(1) (**)	(1) (**)	(2) (*)	(3) (**)
Wong	J Arthroplasty	2018	**	*	*	*	*	**	*	*
Veronica	AOTS	2020	**	*			*	**		

### Clinical outcomes

**Fig. 2:**
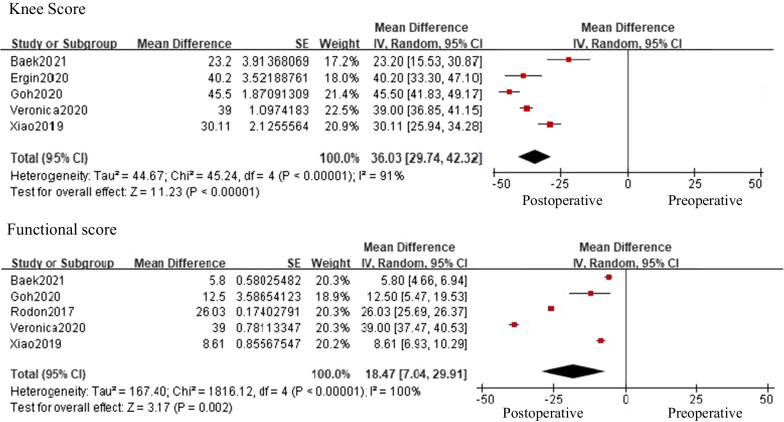
Forest plot of the pooled analysis of knee scores and functional scores before and after total knee arthroplasty in patients with Parkinson’s disease

**Fig. 3:**
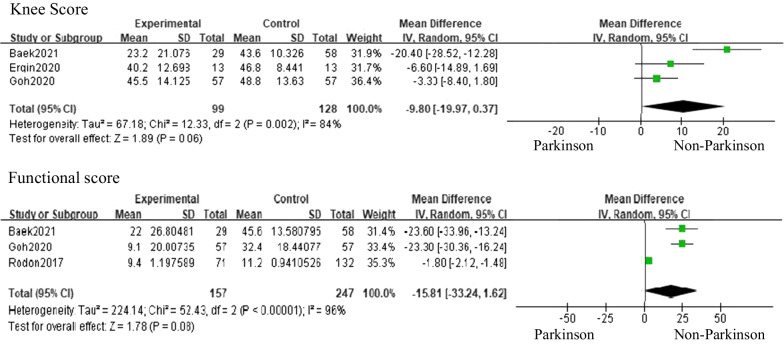
Forest plot of the pooled analysis of improvement in knee scores and functional scores between patients with and without Parkinson’s disease

Nine studies in this review reported on clinical outcomes of TKA in patients with PD (Table 3). Pooled analysis of clinical outcomes was performed for comparison before and after TKA in PD patients and before and after TKA between patients with and without PD (Figs. 2 and 3). The meta-analysis was performed using only studies that reported the mean value and standard deviation of the knee score results. TKA significantly increased knee and functional scores patients with PD (knee score 95% CI: 29.74 to 42.32, with high heterogeneity *I*^2^ = 91%, *p* < 0.00001, functional score 95% CI: 7.04 to 29.91, with high heterogeneity *I*^2^ = 100%, *p* = 0.002), However, compared with knee and functional scores in patients without PD, the increase in scores was not statistically significant in patients with PD, but improvement in patients with PD tended to be less than that in patients without PD (knee score 95% CI: −19.97 to 0.37, with high heterogeneity *I*^2^ = 84%, *p* = 0.06, functional score 95% CI: 33.24 to 1.62, with high heterogeneity *I*^2^ = 96%, *p* = 0.08).

**Table 3:** Clinical outcome comparison after TKA between PD and control patients

	Year	Author	Preoperation (PD)	Preoperation (Control)	Postoperation (PD)	Postoperation (Control)	Others
Acta Orthop. Belg	2013	Craig	KSS 30.5 ROM 95 (30–120)	KSS 32.5 ROM 88 (46–125)	KSS 91 ROM 100 (70–125)	KSS 88 ROM 110 (10–125)	Pain score, ROM were comparable in both group and no functional improvement in PD at postoperative 1 year
J Arthroplasty	2017	Rodon	Functional SF-12 score 27.7 ± 1.06	Functional SF-12 score 32.9 ± 1.14	Functional SF-12 score 37.1 ± 1.67 10 year survival: 66.2%	Functional SF-12 score 44.1 ± 1.27	Improvement in functional scores was significantly less in patients with PD compared to controls (*p* = 0.003) Patients with PD who received TKA demonstrated decreased survivorship than their matched controls (*p* = 0.0045)
J arthroplasty	2018	Wong			OKS 38.3 ± 7.2 ΔOKS 18.6 ± 6.8 ROM 113.2 ± 8.3 ΔROM 12.34 ± 5.4	OKS 40.2 ± 6.5 (*p* = 0.34) ΔOKS 16.5 ± 8.1 (*p* = 0.45) ROM 114.4 ± 9.8 (*p* = 0.62) ΔROM 12.2 ± 4.5 (*p* = 0.96)	
J Orthop Surg Res	2019	Xiao	KSS score Pain 21.11 ± 9.63 ROM 22.22 ± 1.86 Stability 17.89 ± 2.00 Total 61.22 ± 9.66 KSFS 39.72 ± 6.52 SF-12 (PCS) 15.61 ± 3.55 SF-12 (MCS) 20.28 ± 3.49 WOMAC pain 8.39 ± 2.55 WOMAC stiffness 2.94 ± 1.30 WOMAC function 38.00 ± 6.73 ROM: 111.11 ± 9.32		KSS score Pain 43.61 ± 12.10 (*p* < 0.01) ROM 23.61 ± 1.61 (*p* < 0.01) Stability 24.11 ± 1.18 (*p* < 0.01) Total 91.33 ± 12.57 (*p* < 0.01) KSFS 58.06 ± 31.11 (*p* = 0.02) SF-12 (PCS) 20.17 ± 5.08 (*p* < 0.01) SF-12 (MCS) 24.00 ± 5.16 (*p* = 0.02) WOMAC pain 3.39 ± 4.55 (p < 0.01) WOMAC stiffness 0.61 ± 1.50 (*p* < 0.01) WOMAC function 26.44 ± 15.24 (*p* < 0.01) ROM: 118.33 ± 8.22 (*p* < 0.01)		HY I, II stage show better result (KSS total, WOMAC, pain, function) than HY, III, IV and SF-12, WOMAC score correlated with HY stage (*p* < 0.01)
AOTS	2020	Veronica	KSS 32 (20–45) KSFS 34 (28–52) VAS 8 ROM 97		KSS 71 (50–81) KSFS 59 (25–76) VAS 5 ROM 116		
J Arthroplasty	2020	Goh	KSKS 36.5 ± 19.3 KSFS 39.1 ± 24.9 OKS 20.5 ± 10.5 SF-36 PCS 28.8 ± 8.9 SF-36 MCS 45.5 ± 11.5 ROM 108.0 ± 17.7	KSKS 33.1 ± 16.3 KSFS 34.8 ± 25.3 OKS 19.9 ± 9.2 SF-36 PCS 29.3 ± 10.8 SF-36 MCS 47.0 ± 11.6 ROM 105.4 ± 29.2	KSKS 82.0 ± 16.6 KSFS 48.2 ± 26.6 OKS 34.6 ± 10.0 SF-36 PCS 39.6 ± 11.3 SF-36 MCS 50.7 ± 12.2 ROM 110.4 ± 15.1	KSKS 81.9 ± 18.5 (*p* = 0.978) KSFS 67.2 ± 21.4 (*p* < 0.001) OKS 39.0 ± 8.7 (*p* = 0.014) SF-36 PCS 43.5 ± 13.4 (*p* = 0.092) SF-36 MCS 54.1 ± 9.9 (*p* = 0.109) ROM 112.5 ± 17.6 (*p* = 0.490)	
J Orthop Sci	2020	Ergin	KSS 45.4 ± 16.8 ROM 93.9 ± 17.0 Flexion contracture 8.85 ± 7.12	KSS 38.8 ± 11.5 ROM 100.4 ± 14.6 Flexion contracture 7.41 ± 4.84	KSS 85.6 ± 7.60 ROM 99.5 ± 9.37	KSS 85.6 ± 10.0 ROM 109.2 ± 10.2	Similar outcomes compared with general population despite disease severity and progression Mean increase in KSS and ROM values were significantly higher for high-grade patients (*p* < 0.05) High-grade PD: similar outcome
Int Orthop	2021	Baek	KSS 36.8 ± 5.4 Functional score 35.5 ± 4.4	KSS 37.1 ± 4.8 Functional score 35.9 ± 6.7	KSS 60.0 ± 24.5 Functional score 57.5 ± 29.7 Survival rate: 89.7%	KSS 80.7 ± 13.1 Functional score 81.5 ± 17.4 Survival rate: 98.3%	
BMC Musculoskeletal Dis	2022	Y. Zong	EQ-pain and discomfort 2.19 ± 0.60 PDQ-function 57.8 ± 26.3	EQ-pain and discomfort 2.40 ± 0.57 PDQ-function 46.7 ± 21.6	EQ-pain and discomfort 2.37 ± 0.49 PDQ-function 53.3 ± 21.0	EQ-pain and discomfort 1.92 ± 0.58 PDQ-function 32.8 ± 24.3	

KSS: knee society score; KSFS: knee society functional score; KSKS: knee society knee score; MCS: mental component summary; OKS: Oxford knee score; PCSL physical component summary; PD: Parkinson’s disease; ROM: range of motion; TKA: total knee arthroplasty; VAS: visual analog scale; WOMAC: Western Ontario and McMaster Universities Osteoarthritis Index; EQ: Euro QOL; PDQ: Pain and Disability Questionnaire

Two of the included studies reported the results of TKA in patients with PD without a control group [12, 15]. Both studies showed statistically significant improvement in clinical score, pain reduction, and increased range of motion (ROM) after TKA in patients with PD. In another study, postoperative results were divided into a PD patient group and a non-PD patient group, and there was no difference in the Oxford knee score (OKS), ROM after TKA, and the degree of increase in OKS and ROM compared with preoperative values [16]. The remaining five studies compared the preoperative and postoperative clinical outcomes of the PD and non-PD groups. Two of these studies reported that the preoperative and postoperative clinical outcomes did not differ between the two groups [9, 11]. Another four studies reported worse clinical outcomes in the PD group than in the non-PD group after TKA [17–20].

Differences in clinical outcomes according to disease severity after TKA were reported in two studies [11, 12]. Xiao reported that patients with mild PD (Hoehn and Yahr stages I and II) showed better clinical symptom improvement after TKA than patients with severe PD [12]. However, Ergin et al. reported that in patients with high PD severity, the preoperative function was low, but the postoperative function was similar to patients with severe PD [11].

### Complications

Among the studies, 11 studies reported postoperative complications (Table 4). Two studies reported only the complications that occurred in the PD group, which included gastrointestinal disorders, delirium, upper respiratory infection, confusion, and flexion contracture [12, 15]. In another two studies, complications such as infection, urinary retention, and deep vein thrombosis were reported in both groups [9, 16]. However, the number of complications was small, and comparisons were not made. In five studies, the probability of complication occurrence in the PD group was higher than in the non-PD group [1, 18, 19, 21, 22]. The remaining two studies reported no difference between the two groups [8, 23]. Newman et al. reported that delirium, mental status, pneumonia, urinary tract infection, and transfusion rates were higher in the PD group than in the non-PD groups, but there was no difference between the groups in terms of surgical complications [1]. Merchand et al. reported that medical complications, such as transfusion, anemia, cerebrovascular event, and thrombocytopenia, were 3.5 times higher and other implant-related complications, such as loosening and periprosthetic fracture, were 1.6 times higher in the PD group than in the non-PD group [21].

**Table 4:** Complication comparison after TKA between PD and control patients

Journal	Year	Author	PD	Control	Complication
Acta Orthop. Belg	2013	Craig	Deep vein thrombosis, superficial infection, urinary retention, urinary tract infection, quadriceps avulsion, posterior tibial dislocation	Deep infection, urinary retention, patella subluxation, aseptic loosening	
JBJS	2014	Jämsen	PD had no statistical association with the 1-year infection rate at longer follow-up (~5 years), patients with PD had higher mortality [hazard ratio 1.94 (1.68–2.25)]		No difference between PD cases and controls in mortality at 90 days, 180 days, or 1 year
J Arthroplasty	2017	Rodon	Aseptic loosening: three (4.2%) Peri-prosthetic joint infection: nine (12.7%) Arthrofibrosis: three (4.2%) Patella clunk syndrome: none (0%)	Aseptic loosening: one (0.8%) *p* = 0.0909 Peri-prosthetic joint infection: none (0%), *p* < 0.0001 Arthrofibrosis: three (2.3%) *p* = 0.4328 Patella clunk syndrome: eight (6.1%) *p* = 0.0348	
J Arthroplasty	2018	Wong	Deep vein thrombosis: three; superficial wound infection: one	Deep vein thrombosis: one Bowel pseudo-obstruction: one	No difference
J Orthop Surg Res	2019	Xiao			Gastrointestinal disorder: two; delirium: one; upper respiratory infection: one
KSSTA	2019	Newman	Medical complication odds ratio Delirium: 3.57 (2.80–4.54) Altered mental status: 2.46 (1.48–4.09) Pneumonia: 1.35 (1.01–1.81) Urinary tract infection: 1.25 (1.08–1.44) Transfusion of blood: 1.46 (1.34–1.57) Cognitive symptoms, stroke, acute myocardial infarction, pulmonary, gastrointestinal, acute renal failure, pulmonary embolism, deep venous thrombosis: n.s Surgical complication odds ratio Hematoma: 1.01 (0.75–7.35) Wound infection: 1.39 (0.53–3.65) Wound dehiscence: 1.00 (0.32–3.10) Injury–peripheral nerve: 1.50 (0–28-8.19) Irrigation and debridement: 1.42 (0.64–3.14)		
Knee	2019	Kleiner	In-hospital complication rate: 8.3% All complication odds ratio: 1.036 (0.949–1.130) In-hospital mortality: 0.16%	In-hospital complication rate: 8.0% (*p* = 0.4297) In-hospital mortality: 0.15%	Complications were more likely in older patients and patients with increased CCI No difference in complication rate or mortality between PD patients and matched non-PD patients
AOTS	2020	Veronica			Confusion and flexion contracture were the most frequent perioperative complications
JKS	2020	Marchand	Medical complication odds ratio Transfusion rate: odds ratio 6.79 (4.74–9.74) Acute post-hemorrhagic anemia: 4.75 (3.85–5.86) Cerebrovascular accident: 3.48 (2.04–5.94) Thrombocytopenia: 3.37 (1.72–6.62) Urinary tract infection: 3.29 (2.73–3.97) Acute kidney failure: 2.74 (2.00–3.76) Pneumonia: 2.70 (1.72–4.24) Other postoperative infections: 1.67 (1.09–2.57) total medical complications: 3.50 (3.15–3.89) Implant-related complication odds ratio Other mechanical complications: 2.61 (2.13–3.21) Peri-prosthetic fracture: 2.26 (1.70–3.01) Dislocation: 2.22 (1.83–2.69) Broken prosthetic joint implants: 1.79 (1.27–2.53) Peri-prosthetic joint infection: 1.19 (1.04–1.35) Mechanical loosening: 1.33 (1.05–1.69) Revision total knee arthroplasties: 1.55 (0.83–2.89) Total implant-related complications: 1.64 (1.51–1.79)		
J Arthroplasty	2020	Goh	Medical complications Arrhythmia: one; hypotension: three; acute kidney injury: three; urinary tract infection: one; pneumonia 1, delirium 3 Surgical complications: deep infection: one Wound complication: surgical site infection: one; dehiscence one	Medical complication Acute kidney injury: one; acute pancreatitis: one; delirium: two Wound complication: surgical site infection: one; hematoma: one	All complication comparison: *p* = 0.030
J of AAOS	2022	Cheppalli	Odds ratio Blood loss anemia: 1.501 (*p* < 0.001) Peri-prosthetic dislocation: 3.813 (*p* < 0.001) Peri-prosthetic mechanical complication: 1.936 (*p* = 0.024) Mortality: NA(*p* = 0.294) Peri-prosthetic infection: 1.190 (*p* = 0.287)		

CCI: Charlson Comorbidity index; PD: Parkinson’s disease

For detailed complications, the infection rate was reported in five studies [1, 8, 19, 21, 22]. Three registry-based studies reported no difference in infection rate [1, 8, 22]. However, one case–control study and one registry-based study reported that the infection rate was higher in the PD group than in the non-PD group [19, 21]. For aseptic loosening, there was no difference between groups in the study by Rodon, but Merchand reported higher aseptic loosening in the PD group than in the non-PD group. Mortality was reported in three studies [8, 22, 23]. Jämsen et al. reported that the mortality in the PD group was 1.94 times higher than that in the non-PD group at the 10-year follow-up, but Kleiner et al. and Cheppalli et al. reported that there was no difference in mortality between the groups.

## Discussion

The purpose of this systematic review and meta-analysis was to determine the effect of PD on clinical outcomes and complications after TKA. Based on the included studies, the principal findings were as follows: (1) TKA significant improved clinical symptoms in patients with PD and (2) the probability of complications, but not fatal complications, is high in patients with PD after TKA.

Due to the increase in life expectancy, the number of patients with underlying diseases requiring TKA has increased. Moreover, PD is an increasingly common disease in elderly individuals, and it is a disease that lowers the quality of life of patients with gait disorders. It is thought that the outcome of TKA is inevitably affected by PD due to the natural progression of the disease. We believe that PD adversely affects TKA outcomes. However, if the clinical outcomes can be improved significantly, we believe that TKA can be a good treatment for OA with PD, even if the degree of improvement in clinical outcomes is less than that in patients without PD. In this study, most of the included studies showed improved clinical scores in patients with PD after TKA. In addition, four out of six studies showed that the outcomes of TKA were similar between patients with and without PD patients; therefore, the degree of improvement cannot be considered low [9, 11, 16, 18]. Further, the effects of TKA are considered to be different depending on the severity of PD. Two studies compared the clinical outcomes according to PD severity. However, one study reported that there was no difference in clinical outcome according to PD severity, [11] and another study reported that patients with high PD severity had poor outcomes [12]; therefore, this study could not conclude on the effect of PD severity on the clinical outcomes of TKA. In addition, a direct comparison between studies was not possible because the indicators that suggested PD severity in each study were different (Charlson Comorbidity index, Hoehn and Yahr stage, and Elixhauser Comorbidity index).

For medical and surgical complications that may occur after TKA, the definition and inclusion of complications were different for each study, and the probability of complications was also low; therefore, the two groups could not be statistically compared. In four studies included in the analysis, only the types of complications that occurred were presented, but no comparison was made between the two groups [9, 12, 15, 16]. In a registry-based cohort study involving a large number of patients, the risk of medical complications was reported differently [1, 21]. As mentioned above, different risk is thought to be due to the difference in the exact inclusion criteria of the included complications and the difference in the included complications. The probability of pneumonia, transfusion rate, mental status change, and urinary tract infection were high in patients with PD. However, considering that there was no difference in short-term mortality after surgery between the groups, there was no difference in life-threatening medical complications [8, 23].

Regarding surgical complications, the most common concerns are infection rate, aseptic loosening, and revision rate. A small-scale study reported that the probability of infection and loosening was statistically high in patients with PD, but only occurred in < 3–9 cases. [19] In a registry-based cohort study involving a large number of patients, Newman and Merchand’s studies, different opinions were presented on infection and loosening [1, 21]. However, in a study by Merchant, while the probability of surgical complications was high, that there was no difference in the revision rate [21]. Considering this, there seems to be no difference in severe surgical complications requiring revision. This conclusion is consistent with that of a previous meta-analysis on total joint arthroplasty [24].

Our analysis evaluated the effect of PD on TKA outcomes. This study included only patients with or without PD undergoing TKA. In a previous study, total joint arthroplasty in patient with PD was evaluated [24]; however, there was no systematic review or meta-analysis about patients undergoing only TKA. This is considered a strength of this study because there may be differences in results for each arthroplasty. The limitations of this study should also be considered. First, the number of patients was not large in most studies that reported clinical results. In addition, there were differences in the follow-up period. Second, there was no detailed report on the outcome of TKA according to PD severity, and although it affects the TKA outcome, PD severity could not be analyzed. This should be analyzed in future studies. Third, no studies considered the natural progression of PD. In other words, PD itself can cause various medical diseases and reduce the quality of life, and it is necessary to consider them for accurate results. Fourth, other related diseases, rather than PD itself, may have been a confounding factor. Although many of the included studies performed propensity matching for controlling confounding factors, this study itself could not consider the confounding factor due to the nature of systematic review.

## Conclusions

Patients with PD had satisfactory functional improvement and pain reduction after TKA. However, these outcomes were not as good as those in the non-PD group. The PD group had a higher occurrence rate of medical complications than the non-PD group. Further, the PD group had a similar or higher surgical complication rate than the non-PD group.

## Data Availability

The datasets analyzed during the current study are available from the corresponding author on reasonable request.

## Abbreviations

PD: Parkinson’s disease
TKA: Total knee arthroplasty
ROM: Range of motion
OKS: Oxford knee score
